# Prevalence of multiple sclerosis in Croatia: data from national and non-governmental organization registries

**DOI:** 10.3325/cmj.2018.59.65

**Published:** 2018-04

**Authors:** Tomislav Benjak, Vesna Štefančić, Željka Draušnik, Ivan Cerovečki, Dijana Roginić, Mario Habek, Sandra Mihel, Ranko Stevanović

**Affiliations:** 1 Division for Public Health, Croatian Institute of Public Health, Zagreb, Croatia; 2 Alliance of Croatian Multiple Sclerosis Associations, Zagreb, Croatia; 3 Department of Neurology, University Hospital Center Zagreb, University of Zagreb School of Medicine, Zagreb, Croatia; 4 Department of Social Medicine and Epidemiology, University of Rijeka School of Medicine, Rijeka, Croatia

## Abstract

**Aim:**

To update the estimate of multiple sclerosis (MS) prevalence in Croatia using multiple epidemiological tools.

**Methods:**

This level IV, epidemiological study gathered data from three national patient registries and one database of a non-governmental MS patients' organization. Data were extracted on all individuals who had undergone hospital MS treatment, consulted their primary health care providers about MS-related symptoms, been listed as having MS-related disability, or been members of the mentioned non-governmental organization in 2015. A new database was formed, in which all living individuals were identified using a common identification number to prevent double entries. The prevalence rates in 2015 were calculated by age and sex groups.

**Results:**

In total, 6160 patients diagnosed with MS were identified (72% women). Most women with MS were 50-59 years old and most men were 40-49 years old. The overall MS prevalence rate was 143.8 per 100 000 population.

**Conclusion:**

The calculated MS prevalence rate in Croatia in 2015 was more than twice as high as the estimate from 2013. This difference warrants further research into MS epidemiology in Croatia and calls for a rational allocation of funds and human resources to provide adequate care and support to MS patients.

It is difficult to compare multiple sclerosis (MS) incidence and prevalence rates among European countries owing to a lack of epidemiological studies addressing the issue. This has impeded burden of disease assessment and comparative research into the care costs of MS patients (1). The Multiple Sclerosis International Federation published the Atlas of Multiple Sclerosis (Atlas of MS) in collaboration with the World Health Organization (WHO) in 2008 (2) and independently in 2013 (3). These epidemiological publications have facilitated the comparison of epidemiological data across different countries, helping to develop public policies and services for improving the life quality of MS patients. According to the Atlas of MS, the average worldwide MS prevalence was 30 per 100 000 population in 2008 and 33 per 100 000 population in 2013. The rates were reproduced from peer-reviewed articles of local or national epidemiological studies, where possible. Elsewhere, they were taken from local or national patient registries or articles not published in peer-reviewed journals. Regional variations in MS incidence and prevalence based on such reports were significant; some authors ascribe such an unequal distribution to genetic differences, environmental factors (eg, climate), or their interaction (4).

Both Atlas of MS editions list Croatia among the countries with an above-average MS prevalence rate of 60-100 and 20-60 per 100 000 population in 2008 and 2013, respectively. The latter figure implies that patients with MS living in Croatia would number 2500 at most. Moreover, these figures imply that MS prevalence in Croatia decreased in the period 2008-2013, although at that time there were no considerable advances in early detection, diagnostic procedures, treatment, and rehabilitative measures.

In order to better assess the actual MS prevalence in Croatia, we decided to conduct a new study based on data from multiple data sources aggregated in the Croatian Institute of Public Health (CIPH). Per Official Statistical Records Act and the Program of Statistical Activities for the period 2013-2017, CIPH is the national focal point for aggregation of data on health care indicators and their statistical analysis (5,6). In accordance with the WHO recommendations, divisions within the CIPH collect data on recorded disease or disorder cases and related health care measures. However, the number of patients obtained in this way may not represent the overall number of patients with a particular disease, as some patients may choose not to seek health care during a particular time period (eg, when their disease is in remission) or may only seek assistance from a single health care provider (eg, a general practitioner). Recent advances in anonymization techniques have allowed for unambiguous linking of data contained in all CIPH patient registries and databases. These include the Disabled Persons Registry (ROI), containing information an all Croatian citizens diagnosed with professional disability, and the In-patient Statistics Form (BSO) database, containing information on all stationary medical treatments registered in Croatia. Moreover, since 2015 CIPH has been granted the access to the data on primary health care provider services contained in the Central Health Information System of Croatia (CEZIH). Linking these sources allows for the determination of a more accurate number of MS patients, rather than just the pattern of health care services they use.

There have been no previous studies on the MS prevalence of in Croatia using CIPH data. Materljan and Sepčić (7) assessed regional MS prevalence in Croatia, but did not calculate the overall prevalence rate. Moreover, the data they used were largely based on historical reports. The aim of this study was to determine the number of patients currently diagnosed with MS in Croatia, based on data contained in available registries. The hypothesis of this study was that the number of MS patients in Croatia thus determined would differ significantly from the estimations published in Atlas of MS editions.

## Methods

This level IV, epidemiological study included the following data sources:

1) The database containing information on all medical services provided by primary health care providers in Croatia in 2015 (CEZIH)

2) The database containing information on all patients who underwent hospital treatment in Croatia in 2015 (BSO)

3) The Disabled Persons Registry (ROI) of the CIPH

4) The database containing information on all members of the Alliance of Croatian Multiple Sclerosis Associations (SDMSH).

All of the sources listed individuals’ age, sex, residence, and the personal identification number (OIB), serving as a unique identifier. Additionally, the CEZIH database included the International Statistical Classification of Diseases and Related Health Problems (ICD) code attributed to the patient on his or her visit, information about prescribed drugs and referrals, whereas the BSO database included data on the duration of hospital treatment and the associated ICD code. The ROI registry contained information about the extent and cause of the disability (as coded in ICD). The SDMSH database contained no additional information except the demographic data and OIB identifier; in order to acquire SDMSH membership, applicants had to provide a physician’s certificate attesting MS had been diagnosed.

The data analysis was conducted during March and April 2017 in the Division for Public Health of the CIPH. The data were extracted on all individuals who had undergone hospital treatment for MS (BSO database), consulted their primary health care providers for MS-related symptoms (CEZIH database), been listed as having an MS-related disability in the ROI registry, or been members of SDMSH. The inclusion criterion was diagnosed MS, as per the ICD code G35; however, we were not able to evaluate whether every person with the ICD code G35 met the diagnostic criteria for MS, such as McDonald criteria (8). Deceased patients and patients with residence outside Croatia (verified using the national health insurance registry) were excluded from the study.

The data in the tables were extracted from BSO, CEZIH, ROI, and SDMSH databases using SQL Server Management Studio 2012 software (Microsoft, Redmond, WA, USA) and analyzed using Microsoft Excel 2010. All individuals were identified using their OIB number, preventing double entries. Some previous studies have used similar methodology and Eurostat has stated that this method allows for more accurate data collection on MS incidence (9). The study was approved by the CIPH Ethics Committee on March 1, 2017, under the registration number 381-03-351-17-3.

## Results

There were 6160 MS patients; 4394 (72%) were women (women-to-men ratio: 2.49) (Table 1). Most women with MS (n = 1105) were in the age group 50-59, whereas most men (n = 480) were in the age group 40-49. The overall MS prevalence rate in Croatia was 143.8 patients per 100 000 population. The distribution of prevalence rates by age groups was unimodal and roughly normal, with the greatest prevalence rate in the age group 40-49 for both sexes. There was a single MS case in the age group 0-9 (an eight-year old female patient), whereas 72 cases were recorded in the age group over 80; the oldest patient was aged 96 years.

**Table 1:** Patients diagnosed with multiple sclerosis in Croatia by sex and age groups

Age group	Male population		Female population		Total population	
patients	PR*	patients	PR	patients	PR
0-9	0	0.0	1	0.5	1	0.2
10-19	14	5.7	23	9.8	37	7.7
20-29	125	44.5	320	118.6	445	80.8
30-39	415	141.2	856	299.9	1271	219.4
40-49	480	162.1	1052	352.5	1532	257.7
50-59	370	118.7	1105	344.7	1475	233.3
60-69	256	117.9	758	294.3	1014	213.6
70-79	86	55.4	227	97.6	313	80.7
80 +	20	38.9	52	44.4	72	42.7
Total	1766	85.5	4394	198.1	6160	143.8

*PR – prevalence rate per 100 000 population.

†Data on the number of inhabitants in particular sex and age groups (2011 Census) were retrieved from the Croatian Bureau of Statistics web-site (10).

The greatest proportion of MS patients was included in the CEZIH database, accounting for 82.9% of the total number of MS patients; less than 50% was included in other data sources (Table 2). In addition, the CEZIH database included 863 patients not listed in any of the other data sources (14.0% of the total number of MS patients); the respective numbers in other databases were considerably lower. There was a considerable overlap between the data sources, especially with regard to the CEZIH database (Table 3).

**Table 2:** Number of multiple sclerosis (MS) patients in Croatia included in individual data sources

Data source	Number of MS patients in the respective database	Proportion of total number (%) of MS patients (N = 6160)	Number of MS patients listed only in respective database	Proportion (%) of MS patients listed only in the respective database (N = 6160)
Central Health Information System of Croatia	5106	82.9	863	14.0
In-patient Statistics Form database	2966	48.1	187	3.0
Disabled Persons Registry	2962	48.1	285	4.6
Alliance of Croatian Multiple Sclerosis Associations	2665	43.2	100	1.6

**Table 3:** Number of multiple sclerosis (MS) patients in Croatia included in multiple data sources*

Number of databases	Number of MS patients included in listed databases	Proportion (%) of MS patients included in listed databases (N = 6160)
Two databases*		
CEZIH + BSO	1135	18.4
CEZIH + ROI	467	7.6
CEZIH + SDMSH	247	4.0
BSO + ROI	34	0.6
BSO + SDMSH	27	0.4
ROI + SDMSH	180	2.9
Three databases		
CEZIH + BSO + ROI	321	5.2
CEZIH + BSO + SDMSH	436	7.1
CEZIH + ROI + SDMSH	849	13.8
BSO + ROI + SDMSH	38	0.6
Four databases		
CEZIH + BSO + ROI + SDMSH	788	12.8

*CEZIH – Central Health Information System of Croatia; BSO – In-patient Statistics Form database; ROI – Disabled Persons Registry; SDMSH – Alliance of Croatian Multiple Sclerosis Associations.

## Discussion

The total number of MS patients in Croatia determined in this study exceeds manifold the estimations in the 2008 and 2013 Atlas of MS editions. Considering the derived MS prevalence rate, Croatia groups among the countries with the highest disease prevalence (prevalence rate higher than 100 per 100 000 population). This group includes the United States, Canada, the United Kingdom, and the Scandinavian countries (3,11). A number of previous studies purported there was an MS prevalence south-north gradient, with the highest rates in Northern European population; however, the study by Wade and the results of our study suggest there are other pathogenetic factors that should be taken into consideration (12).

Planning and policy design in the health care system, key to achieving optimal outcomes in patient care, should be based on accurate information (13). Had resource planning and allocation in Croatia been done based on the Atlas of MS data, erroneous conclusions and inadequate health care provision would have been inevitable. During the preparation of both Atlas editions, the data-gathering coordinators for Croatia were SDMSH members and their associates. They derived the required MS epidemiology indicators from the SDMSH member registry: more specifically, the number of SDMSH members was used as the approximate number of MS patients in Croatia. However, membership in SDMSH is purely voluntary; the necessary prerequisites are the clinically confirmed MS diagnosis (ICD code: G35) and an established residency in Croatia. Our results show that many MS patients were not included in the SDMSH registry, and that other data sources, CEZIH in particular, allow for more exact estimates to be made.

Although data from four different sources were used and cross-linked in this study, allowing for greater accuracy, it is possible that the prevalence of MS in Croatia may be even higher, because not all patients may have sought medical assistance for MS in national health care institutions during 2015, nor were all MS patients obliged to be members of SDMSH. Moreover, there is a possibility that in some situations the ICD code G35 may have been misattributed.

The United Kingdom National Institute for Health and Care Excellence (NICE) recommends that the period between the onset of MS symptoms and the first neurological referral examination should not exceed six weeks (14). Also, the period between the first examination and completion of all diagnostic tests should not exceed six-weeks. According to the 2013 study, only 61.5% of patients diagnosed with MS in Croatia were referred to a neurologist within six weeks from the symptoms' onset, whereas only 64.1% were diagnosed with MS during the following six weeks (15). The discrepancy between the current practice in Croatia and the NICE recommendations might be caused by a lack of neurologists and other necessary personnel involved in neurological care, as the number of patients far exceeded the one expected, in addition to limited financial and material resources. The failure to meet the NICE recommendations (for over a third of all patients) is unsurprising if we take into consideration that previous health care provision plans had been based on the estimate of 2500 MS patients living in Croatia.

However, the main reason why our results could significantly influence MS patients' medical care is that an early referral to specialized MS center can improve outcomes (16), since early referral leads to earlier diagnosis and earlier treatment. With the changing landscape of treatment options for MS and available therapies that can alter the disease course, the knowledge about the scope of the problem will enable health care authorities to better plan resources needed for appropriate management of MS patients.

Regarding direct expenses of care in the United States, MS ranks as the second most costly chronic disease (behind congestive heart failure) (17). It is reasonable to assume that treatment costs and burden of disease grow as the patient's disability progresses. However, recent studies from the United States warn of a lack of current data on the effect of MS-related disability on treatment expenses (18,19). In Croatia, there are no systems comprehensively monitoring medical expenses related to particular medical entities, including MS. Considering the current health care system reform proposals, the data presented in this research may help in future planning and allocation of financial health care resources for MS patients in Croatia.

The study's limitation are the possible errors in the use of ICD code G35, designating MS in BSO and CEZIH databases. The database used in this study was formed by aggregating data entries from different databases, rather than by directly examining patient histories. However, the influence of misattribution errors is likely to be small and could not have significantly altered the results. Data entries extracted from the ROI and SDMSH databases were attested for by medical records.

The current MS prevalence rate in Croatia is 143.8 patients per 100 000 population, greatly exceeding previously published formal estimates. By linking anonymized data extracted from multiple databases we determined a revised prevalence rate, which may help health care providers in Croatia to amend plans on resource allocation and better conform to NICE MS guidelines. To further improve epidemiological assessments and consequent resource planning, it would be beneficial to establish a national registry of MS patients. Furthermore, BSO and CEZIH databases contain some additional information not analyzed in this study. It may be useful to analyze these data and present them to the decision makers. Conducting similar studies to determine the prevalence of other common diseases may also be of benefit.
